# Simultaneous subarachnoid hemorrhage and cerebral infarction due to aneurysm rupture and occlusion of the parent terminal artery: A cause of angiogram-negative non-perimesencephalic subarachnoid hemorrhage

**DOI:** 10.1007/s10143-025-03957-5

**Published:** 2025-12-11

**Authors:** Shadi Al-Afif, Heinrich Lanfermann, Yannick A. Wilken, Harold F. Hounchonou, Marie Middendorff, Joachim K. Krauss, Omar Abu-Fares

**Affiliations:** 1 Department of Neurosurgery, Hannover Medical School, Carl-Neuberg-Straße 1, 30625 Hannover, Germany; 2 Institute of Diagnostic and Interventional Neuroradiology, Hannover Medical School , Hannover, Germany

**Keywords:** Basilar artery perforator aneurysm, Lenticulostriate artery aneurysm, Negative angiogram, Non-perimesencephalic, Subarachnoid hemorrhage, Lacunar infarction

## Abstract

Spontaneous subarachnoid hemorrhage (SAH) is most often caused by rupture of an intracranial aneurysm. However, in approximately 15–20% of cases, no clear source of bleeding can be identified. While angiogram-negative perimesencephalic SAH is characterized by localized blood clot distribution and is generally associated with favorable outcome, angiogram-negative non-perimesencephalic SAH presents a more diffuse blood pattern resembling aneurysmal hemorrhage and is linked to worse outcome, and its causes and etiology remain unclear. We hypothesize that rupture of small terminal artery aneurysms followed by parent vessel occlusion may account for a subset of angiogram-negative non-perimesencephalic SAH. Over a 10-year period, we reviewed the imaging studies of 524 patients with SAH admitted to our institution to identify patients with angiogram-negative non-perimesencephalic SAH. Clinical data, imaging findings, and outcomes were analyzed. Follow-up data were supplemented prospectively. Seventy two patients (14%) were identified with angiogram-negative SAH. Of these, 45 patients (63%) presented with perimesencephalic SAH, while 27 patients (37%) had non-perimesencephalic patterns on CT imaging. Among the non-perimesencephalic group, 9 patients (33%) exhibited small brain infarctions correlated with parent artery occlusion. These patients (mean age 68 years) presented with severe SAH (Fisher grade 3 or 4) and Hunt and Hess grades ranging from 2 to 4. Chronic arterial hypertension was present in 8 of 9 cases. All patients required cerebrospinal fluid diversion, and two developed vasospasm. At follow-up, 5 patients had a good outcome, 2 had moderate disabilities, and 2 patients were dead. This series suggests that ruptured aneurysms of small terminal arteries combined with occlusion of these arteries may cause angiogram-negative non-perimesencephalic SAH. In these patients, characteristic localized infarcts in the territory of the occluded terminal artery support this mechanism. The clinical outcome in these cases is similar to that observed in aneurysmal SAH, underscoring the need for diligent management.

## Introduction

Spontaneous subarachnoid hemorrhage (SAH) is most often caused by the rupture of a saccular aneurysm. In less frequent cases, it can result from ruptured arteriovenous malformations, arterial dissections, tumors, or other vascular pathologies [1]. However, approximately 15–20% of spontaneous SAHs have no identifiable sources [2, 3]. Perimesencephalic SAH confined to the perimesencephalic or prepontine regions accounts for 20 to 70% of angiogram-negative cases and is generally associated with good clinical outcome [4]. Angiogram-negative non-perimesencephalic SAH, however, often is more widespread and may resemble aneurysmal bleeding patterns associated with more frequent complications and worse outcome [5–8].

The differences in blood distribution on initial cerebral CT scans and in the clinical severity between perimesencephalic and non-perimesencephalic SAH indicate distinct etiologies. Whereas perimesencephalic SAH is often linked to venous bleeding, [9] the cause of non-perimesencephalic SAH remains unclear. The more severe clinical presentation of non-perimesencephalic SAH, however, strongly points to arterial sources and it has been assumed that the underlying aneurysm may have been too small to be detected by angiography or it may have been obscured by thrombosis or vasospasm [10].

As aneurysms can also arise on small terminal brain arteries, [11–14] we hypothesize that a certain subset of angiogram-negative non-perimesencephalic SAHs may result from the rupture of such aneurysms on the short segment of a terminal artery located within the subarachnoid space before its entry into the brain parenchyma. Following rupture, thrombosis or dissection of the terminal artery may lead to its occlusion, making the aneurysm undetectable on angiographic studies. Given that terminal arteries are end-organ vessels, the occurrence of a singular and localized brain infarction after SAH provides evidence supporting parent terminal artery occlusion caused by aneurysm rupture [15–18]. To better characterize such events and to determine their frequency, we analyzed the imaging studies of patients who presented to our clinic with angiogram-negative SAH over a 10-year-period.

## Methods

From December 2014 to November 2024, a total of 524 patients were admitted with SAH to the Department of Neurosurgery at Hannover Medical School. Imaging studies, including CT, CT-angiography, and transfemoral digital subtraction angiography of the carotid and the vertebral arteries were reviewed retrospectively by a board-certified neuroradiologist and a board-certified neurosurgeon.

Inclusion criteria for the present study were: 1-non-perimesencephalic SAH on the initial CT as described by van Gijn et al. (widespread distribution of subarachnoid blood, extending beyond the perimesencephalic cisterns, especially in the chiasmatic cistern, the interhemispheric fissure, the lateral part of the Sylvian fissures, and the suprasellar cistern); [10, 19] and 2-detection of brain infarction related to occlusion of a terminal artery on follow-up imaging studies. Patients who had a readily identifiable cause for bleeding on initial and follow-up imaging studies were excluded.

Subsequently, demographic, clinical, and follow-up data of the included patients were collected retrospectively and partially prospectively through telephone interviews. The outcome at follow-up was measured using the modified Rankin Scale (mRS) which is widely used for measuring the degree of disability or dependence in daily activities of people who have experienced a stroke or other neurological conditions [20]. The mRS ranges from 0 to 6 (0: no symptoms; 1: no significant disability, able to carry out all usual activities, despite some symptoms; 2: slight disability, able to look after own affairs without assistance, but unable to carry out all previous activities; 3: moderate disability, requiring some help, but able to walk without assistance; 4: moderately severe disability, unable to attend to own bodily needs without assistance and unable to walk unassisted; 5: severe disability, bedridden, incontinent, and requiring constant care; 6: death.

## Results

**Fig. 1:**
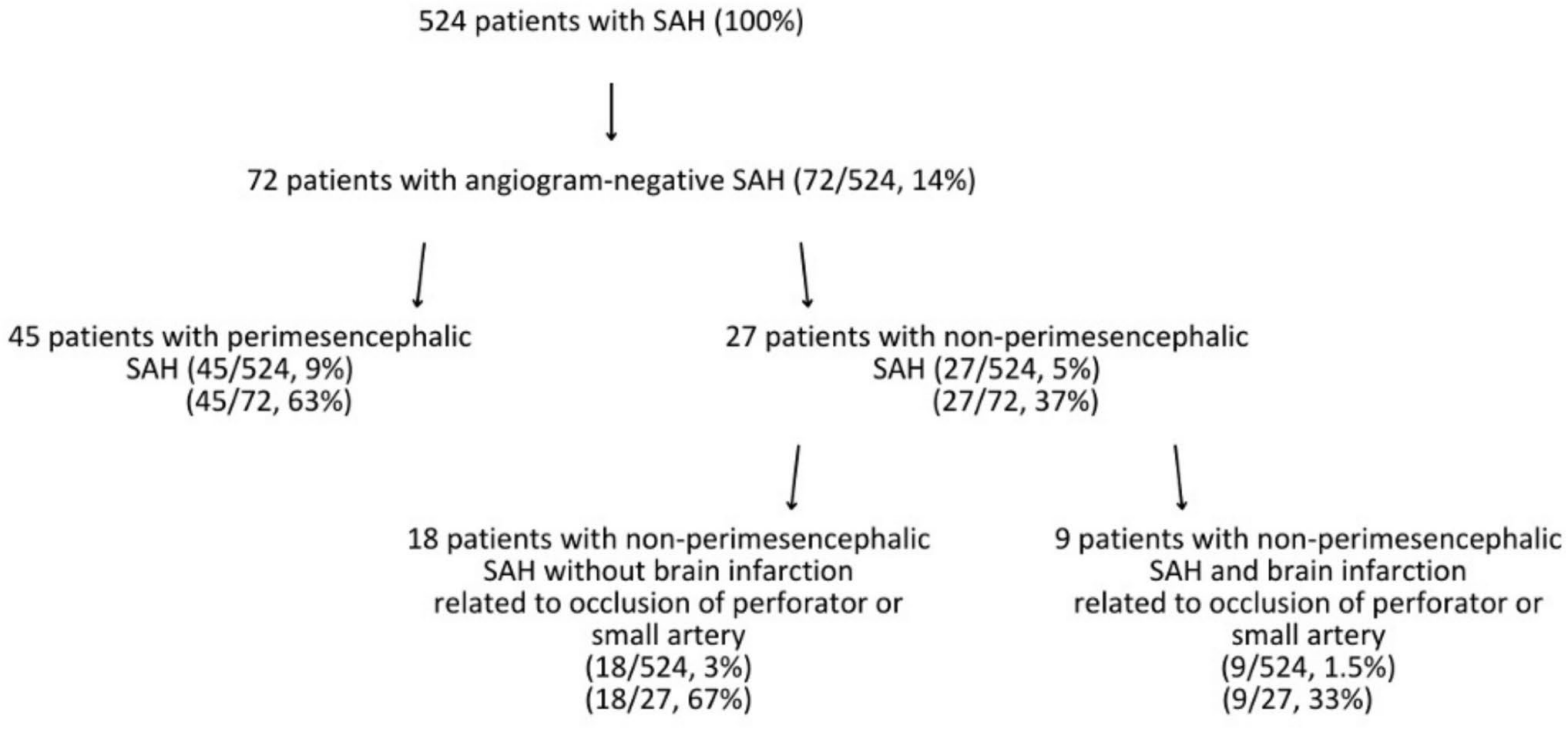
Distribution based on characteristics and causes of subarachnoid hemorrhage

Of the 524 patients presenting to our department with SAH, 72 patients (14%) had no evidence of an aneurysm on angiography or any other identifiable cause for SAH (angiogram-negative) (Fig. 1). Among these, 45 patients (63%) exhibited a typical perimesencephalic SAH pattern on their initial CT scan. The remaining 27 patients (37%) had a non-perimesencephalic SAH pattern on the initial CT resembling an aneurysmal bleeding (Fig. 1).

From the 27 patients with non-perimesencephalic SAHs, 9 patients (33%) had brain infarctions on the following imaging studies (6 detected on MRI and 3 on CT), which were related to occlusion of a terminal artery but unrelated to vasospasm.

### Clinical and demographic data

The patient’s age ranged from 56 to 83 years (mean 68 years). Five patients were female, four patients were male. Common presenting symptoms included headache, nausea, vomiting, and nuchal rigidity (Table 1). According to the Hunt and Hess scale, the severity of clinical presentation ranged from grade 2 to grade 4, reflecting varying degrees of neurological impairment at admission. Arterial hypertension was a prevalent comorbidity (8/9 patients), along with other conditions such as hyperlipidemia, obesity, diabetes type 2, and coronary heart disease (Table 1). Based on initial CT imaging, five patients were classified as Fisher grade 4 and four as Fisher grade 3, indicating the presence of a significant volume of subarachnoid blood (Table 1).

**Table 1:** Clinical and demographic data of 9 patients with non-perimesencephalic SAH and related brain infarction. The table summarizes age, sex, presenting symptoms, presence of arterial hypertension, relevant medical history, and grading according to Fisher grade (CT) and Hunt and Hess garde

Patient No	Age	Sex	Symptoms at presentaion	Arterial hypertension	Comorbidities	Fisher grade on CT	Hunt and Hess grade
1	83	F	nausea, vomiting, lethargy	yes	HLD, FFx	4	4
2	58	F	headache, nausea, vomiting, obtundation	yes	-	3	3
3	67	M	headache, nausea, vomiting, hemiparesis, obtundation	yes	-	3	3
4	56	F	headache, neck pain, nausea, vomiting, drowsiness	no	BC, BCC	3	2
5	57	F	headache, meningism, dysphagia, hemiparesis, obtundation	yes	-	4	3
6	71	F	headache, meningism, nausea, vomiting	yes	OB, T2D	3	2
7	61	M	headache, meningism	yes	HC	4	2
8	82	M	coma	yes	CHD, CAU, LC, OB, T2D	4	4
9	75	M	headache, agitation	yes	PC	4	2

*BC* Breast cancer, *BCC* Basal-cell carcinoma, *CAU* Chronic alcohol use, *CHD* Coronary heart disease, *FFx* femoral fracture, *HC* hypercholesterolemia, *HLD* hyperlipidemia, *LC* Liver cirrhosis, *OB* Obesity, *PC* Prostate cancer, *T2D* Type 2 diabetes

### Imaging data and clinical course

**Fig. 2:**
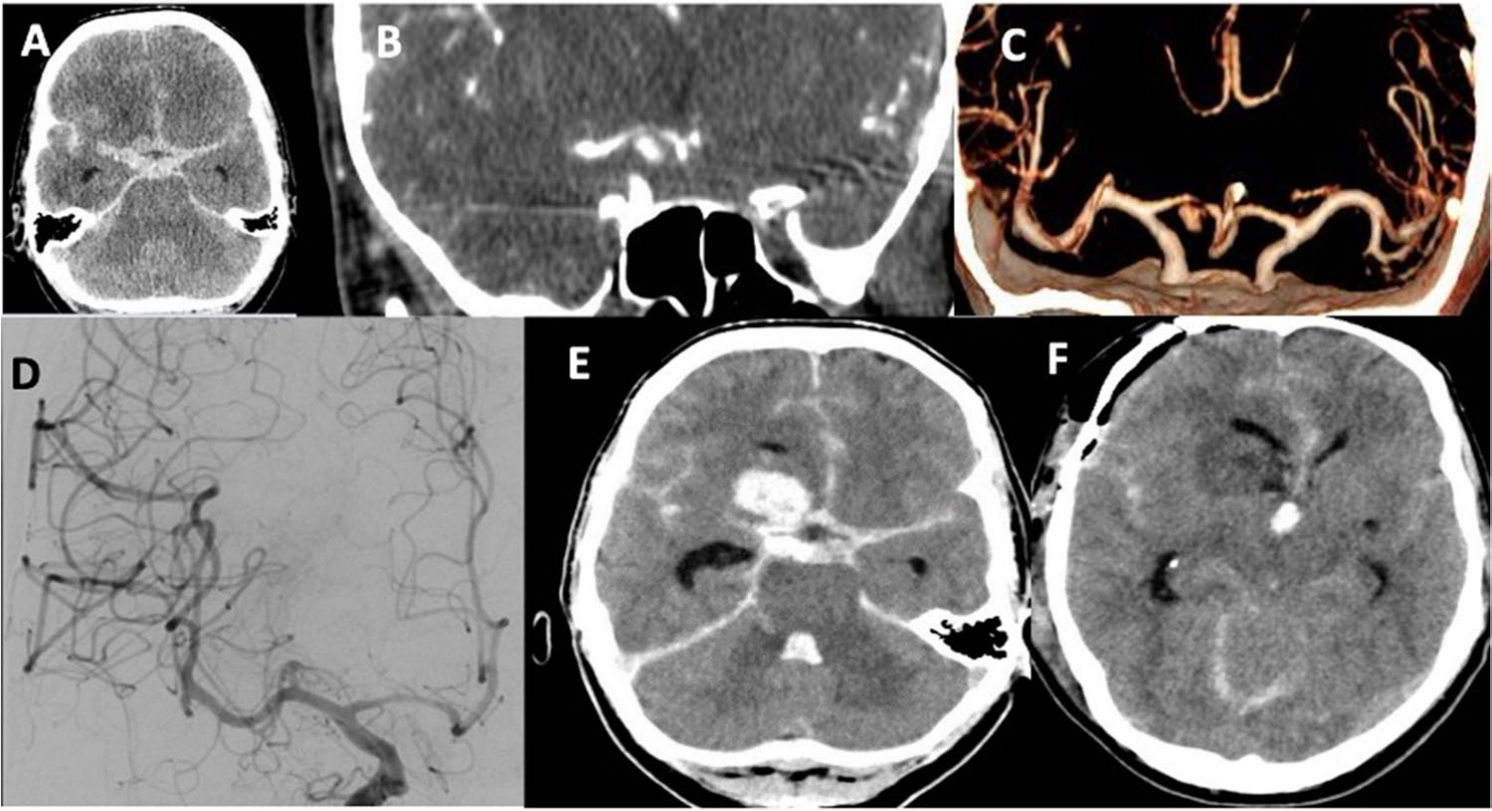
Case 1, 58-year-old woman. Computed tomography (CT) with axial reconstructions (**A**) performed immediately after the onset of severeheadache revealed a subarachnoid hemorrhage, Fisher grade 3, caused by the rupture of an aneurysm related to the right anterior cerebral artery (**B**, **C**). Transfemoral digital subtraction angiography (**D**), performed at the same day of symptoms onset, did not show the aneurysm. A control CT (**E**) immediately after the angiography revealed a new intracerebral bleeding in the right frontal lobe with intraventricular hematoma. The patient underwent emergency surgery, in which a large thrombosed fusiform aneurysm of Heubner’s artery with total occlusion of the parent artery was detected. Follow-up CT scans (**F**) showed an infarction in the right caudate nucleus near the frontal horn of the right lateral ventricle due to occlusion of Heubner’s artery
- Case 2 (Fig. 3): A 67-year-old man with a history of arterial hypertension was admitted with severe headache, nausea and vomiting followed by right-sided hemiparesis und decreased consciousness. A cranial CT scan and CT-angiography (not shown) at admission revealed a SAH, Fisher grade 3, more prominent on the left than on the right side, with a prepontine blood clot in addition, without evidence of an aneurysm, accompanied by acute hydrocephalus. The patient underwent emergency placement of an EVD via a right frontal burr hole trepanation. On the same day, a transfemoral cerebral panangiography was performed which showed no evidence of a ruptured aneurysm. He was admitted to the neurointensive care unit, where he remained intubated and sedated. Five days after admission, he was successfully extubated. One week after the event, he presented with aphasia, prompting another cerebral angiography that revealed vasospasms in both middle cerebral artery territories. He underwent intraarterial spasmolysis using Nimodipine, which was repeated two more times. An MRI two weeks after the event revealed a small pontine infarction on the left side which was related to an occlusion of a perforator of the basilar artery. One month after the event, a control cranial CT scan clearly showed demarcation of the small left-sided pontine infarction. Five months after transfer to rehabilitation, the patient suffered from moderate disability, requiring some assistance but able he was able to walk unassisted.

In all 9 patients, the initial and repeated transfemoral angiographies failed to demonstrate an aneurysm or other cause of SAH. The imaging and clinical data are shown in Table 2. In three instances (Patients 1, 2, and 7) aneurysms arising from terminal arteries were identified on the initial CT angiography performed prior to transfemoral angiography; however, panangiographies failed to demonstrate the aneurysm due to occlusion of the parent terminal artery (see Figs. 2). In two instances (patients 2 and 5), the SAH was accompanied by intracerebral hematoma (see Fig. 2). In one instance (patient 2), the patient underwent surgical evacuation of the intracerebral hematoma, and a fusiform aneurysm along with occlusion of the parent artery (Heubner artery) was confirmed intraoperatively using intraoperative Doppler sonography and indocyanine green (ICG) angiography.

**Table 2:** Imaging and clinical course of 9 patients with non-perimesencephalic SAH and related brain infarction. The table presents the presence of aneurysms on initial CT angiography, infarct and hematoma location, the occluded parent artery, number of DSAs performed, occurrence of vasospasm, type of CSF drain, need for VP shunt, and duration of ICU stay

Patient No	Aneurysm on the intial CT-Angiography	Location of Infarct	Location of hematoma	Perforator or small vessel occluded	Number of DSAs	Vasospasm	Type of CSF drain	VP shunt	Duration of ICU stay in days
1	yes	superior semilunar lobule of cerebellum	-	small branch of the PICA	2	no	EVD	no	29
2	yes	head of caudate nucleus	frontobasal	Heubner’s artery	2	no	EVD	yes	29
3	no	ponto- mesencephalic border	-	basilar perforator artery	5	yes	EVD	no	29
4	no	posterior putamen	-	lateral lenticulostriate artery	2	no	EVD	no	16
5	no	medial part of cuneus	occipital lobe	small branch of the parietooccipital artery	2	no	EVD	no	22
6	no	parahippocampal gyrus	-	small branch of anterior choroidal artery	2	no	LD	no	16
7	yes	anterior limb of inferior internal capsule	-	lateral lenticulostriate artery	3	no	LD	no	11
8	no	posterior ventrolateral thalamus	-	thalamoperforating artery	2	yes	EVD	no	34
9	no	cingulate cortex	-	small branch of pericallosal artery	2	no	EVD	yes	25

*CSF* Cerebrospinal fluid, *CTA* Computed tomography angiography, *DSA* Digital subtraction angiography, *EVD* External ventricular drain, *ICH Intracerebral* hematoma, *ICU* Intensive care unit, *LD* Lumbar drain, *MRA* Magnetic resonance angiography, *VP shunt* Ventriculo-peritoneal shunt

In all 9 patients, the small infarctions which were demonstrated on the imaging studies, were related to occlusion of a terminal artery. The sites of infarcts included the cerebellum, caudate nucleus, pons, thalamus, and cingulate cortex (Table 2). The corresponding vessels were all end-organ vessels including Heubner’s artery, a basilar perforator artery, lenticulostriate arteries, a thalamoperforating artery, and small branches of other arteries (Table 2).

All patients underwent cerebrospinal fluid drainage with external ventricular drains (EVD) used in seven cases and lumbar drains in two cases. Two patients required ventriculoperitoneal shunts for subsequent hydrocephalus. Vasospasms occurred in two patients, with one requiring therapeutic intervention by intraarterial vasospasmolysis using Nimodipine. The number of control digital subtraction angiographies ranged from two to five per patient. ICU stay varied between 11 and 34 days, depending on the severity of the clinical course and complications.

### Outcome and follow-up data

The clinical outcomes of the 9 patients with non-perimesencephalic, angiogram-negative SAH demonstrated significant variability (Table 3). At discharge, a wide range of deficits was observed, with some patients experiencing severe impairments such as impaired consciousness, aphasia, dysphagia, hemiparesis, and urinary incontinence (Table 3). Others exhibited minimal or no symptoms. The mRS at discharge ranged between 0 and 5. While three patients had mRS scores between 0 and 1, six patients had scores between 3 and 5, reflecting moderate to severe disability.

**Table 3:** Outcome data of 9 patients with non-perimesencephalic SAH and related brain infarction. The table summarizes the clinical symptoms and modified Rankin scale (mRS) at discharge, duration of follow-up, and mRS at last follow-up

Patient No	Symptoms at discharge	mRS at discharge	Last FU in months	mRS at last FU
1	impaired consciousness, aphasia, dysphagia, urinary incontinence	5	1	6
2	hemiparesis, dysphagia, urinary incontinence	5	5	2
3	hemiparesis, aphasia, dysphagia	4	4	3
4	-	0	3	0
5	lower limb paresis, dysphagia, urinary Incontinence	5	6	3
6	headache	1	17	1
7	-	0	2	0
8	coma	5	-*	6
9	desorientation	3	16	0

*FU* Follow-up, *mRS* Modified Rankin scale, *PMR* Psychomotor retardation, *SAH* Subarachnoid hemorrhage

^*^Patient deceased before follow up

Follow-up periods ranged from 2 to 17 months. At follow-up, outcomes varied notably. Two patients had died. Seven patients had mRS scores between 0 and 3. Four patients showed improvement, with lower mRS scores and returning to independent functionality. Three patients, however, had persistent deficits, including motor impairments and cognitive dysfunction.

#### Case illustrations

- Case 1 (Fig. 2): A 58-year-old woman with a history of arterial hypertension presented with sudden onset of severe headache, nausea and vomiting. She was initially admitted to a local neurology department, where a cranial CT scan revealed a SAH, Fisher grade 3, with suspicion of a ruptured aneurysm related to the right anterior cerebral artery on CT-angiography. The patient’s condition rapidly deteriorated, leading to a loss of protective reflexes and a subsequent intubation was necessary. She was transferred to our clinic for further management. Upon arrival, the patient was intubated and ventilated. An emergency EVD was placed via a left frontal burr hole to manage acute hydrocephalus. A digital subtraction angiography performed on the day of admission revealed no aneurysm. A cranial CT performed immediately afterwards showed a new intracerebral bleeding in the right frontal lobe with intraventricular hematoma. The patient underwent emergency surgery, which revealed a thrombosed fusiform aneurysm of Heubner’s artery with no blood flow in the parent artery. The aneurysm was clipped, and the hematoma was evacuated. Postoperatively, the patient was transferred to the neurocritical care unit. Transcranial Doppler showed no signs of vasospasm. The patient was successfully extubated 18 days after surgery. Follow-up CT scans revealed an infarction in the right caudate nucleus related to occlusion of Heubner’s artery on the right side. Subsequent angiographic studies confirmed complete exclusion of the aneurysm and no evidence of vasospasm. During her intensive care stay, a ventriculoperitoneal shunt was placed. At the time of her transfer to rehabilitation, 30 days after surgery, she was awake and able to follow commands. Two months after surgery she was independent and oriented.

**Fig. 3:**
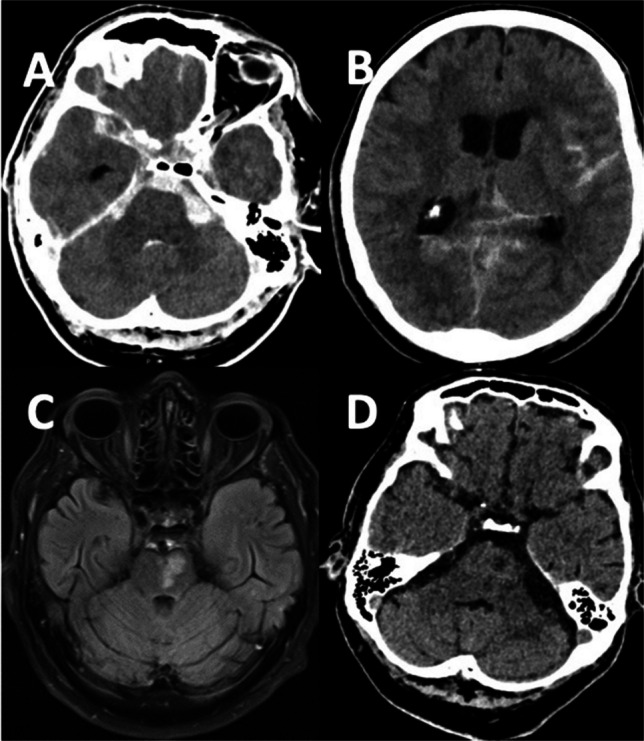
Case 2, 67-year-old man. Computed tomography (CT) with axial views (**A**, **B**), performed immediately after the onset of symptoms including severe headache nausea, vomiting, and loss of consciousness, revealed a subarachnoid hemorrhage, Fisher grade 3, with a large prepontine blood clot, more prominent on the left side than on the right side, accompanied by acute hydrocephalus, without evidence of an aneurysm on CT-angiography or digital subtraction angiography performed on the same day (not shown here). Axial FLAIR MRI sequences obtained after the event revealed a small left-sided infarction on the ponto- mesencephalic border (**C**), likely due to the occlusion of a small basilar artery perforator supplying the brainstem
- Case 3 (Fig. 4): A 56-year-old woman presented with sudden onset of severe bifrontal headache, neck pain, sweating, and nausea. A cranial CT scan revealed an extensive SAH, Fisher grade 3. CT-angiography did not show any cerebral aneurysm. Upon arrival, the patient was intubated and ventilated due to a deterioration in her neurological status. Transfemoral pangiography on the same day did not reveal an aneurysm or another bleeding source. An emergency EVD was placed via a right frontal burr-hole to treat hydrocephalus, and the patient was admitted to the neurocritical care unit. She was extubated the next day, and no neurological deficits were observed. Daily transcranial Doppler ultrasounds showed no evidence of vasospasm. An MRI performed 10 days after the event revealed a small infarction in the left posterior putamen, likely caused by the occlusion of a small perforator artery from the lateral lenticulostriate branches of the middle cerebral artery on the left side. Clinically, the patient remained without neurological deficits. A follow-up angiography performed two weeks after the initial presentation did not identify any bleeding source. The EVD was successfully removed without the need for the placement of a ventriculoperitoneal shunt. She remained neurologically stable throughout her stay. By the time of discharge, 20 days after the admission, the patient was awake, fully oriented, in good general condition, and her neurological examination was unremarkable.

**Fig. 4:**
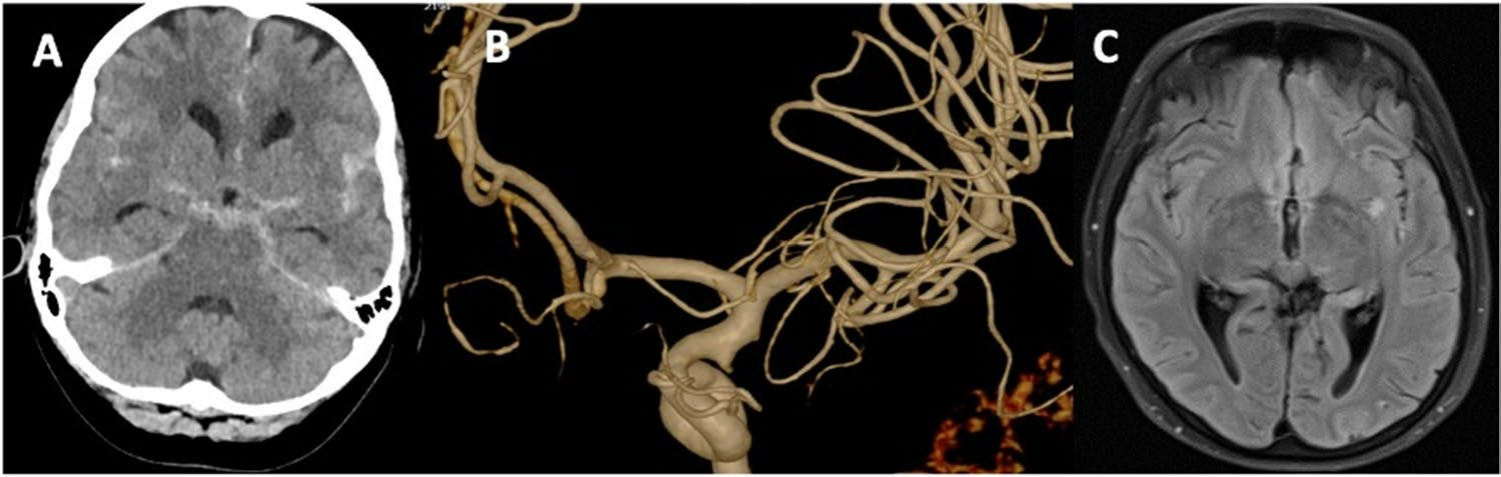
Case 3, 56-year-old woman. Computed tomography (CT) with axial views (**A**), performed immediately after the onset of severe bifrontal headache, neck pain, sweating, and nausea, revealed a subarachnoid hemorrhage, Fisher grade 3, with more dominant blood distribution in the left Sylvian fissure then in the right one, without evidence of an aneurysm on CT-angiography (not shown here) or on transfemoral digital subtraction angiography (**B**) performed on the same day. Axial FLAIR MRI sequences (**C**) revealed a small left-sided infarction in the left posterior putamen due to the occlusion of a small perforator from the lateral lenticulostriate artery group, suggesting the rupture of an aneurysm located on this vessel

## Discussion

Our present systematic study reviewing a large database of 524 patients indicates that acute SAH resulting from the rupture of an aneurysm located on a small terminal artery may be more frequent than suggested by occasional case reports. In fact, it may account for 1.5% of SAH, comprising as much as 33% of angiogram-negative non-perimesencephalic SAH. While a definite proof was available only in 3 patients of our series of 9 patients, there is indirect evidence considering that the occurrence of infarction of the aneurysm-bearing artery results in disappearance of the aneurysm.

The actual frequency of angiogram-negative non-perimesencephalic SAHs resulting from the rupture and occlusion of an aneurysm of a terminal artery might even be higher in angiogram-negative SAH for several reasons. First, not all patients in our study with non-perimesencephalic SAH underwent MRI to detect infarctions, and small infarctions, particularly in the infratentorial space, may have been missed on CT scans.

Second, some patients with multiple infarctions related to vasospasm after non-perimesencephalic SAH were not included in our study, even though a subset of these cases might have been caused by infarctions due to parent artery occlusion after aneurysm rupture.

Third, not all aneurysm ruptures of small arteries result in infarction, as the vessel lumen may remain patent, and only the small aneurysm undergoes thrombosis and occlusion [21]. On the other hand, anatomical studies have shown that certain perforating vessels form anastomoses in their cisternal segments. These anastomoses are more frequently observed among the thalamoperforating branches of the PCA, as well as among the anterior lenticulostriate arteries, [22] and they may prevent the development of distal infarction when a proximal cisternal artery segment is occluded.

As early as in the mid 1980 s, Van Gijn and colleagues distinguished two types of negative-angiogram SAH with different clinical outcomes: perimesencephalic and non-perimesencephalic SAH [19]. This distinction was based on the distribution of blood observed in the initial cerebral CT performed within the first 24 h after the clinical event.

Perimesencephalic SAH is characterized by blood localized in the perimesencephalic cisterns anterior to the brainstem, which may extend to the ambient cisterns and the basal parts of the Sylvian fissures. In contrast, the non-perimesencephalic SAH pattern exhibits a more diffuse blood distribution that goes beyond the aforementioned regions [10, 19].

Similar scenarios as reported in our study have been described previously in the form of few case reports, [14, 21, 23] but no systematic investigation has been available thus far. Our group has reported earlier on a patient with SAH secondary to lenticulostriate artery aneurysm causing SAH which was detectable on CT-angiography but was not shown on transfemoral angiography since thrombosis occurred within hours resulting in infarction [24].

In the patients of the present series, the causes of the occlusion of the parent artery may include thrombosis or dissection of the artery. The form of the aneurysm may determine whether the parent vessel becomes occluded when the aneurysm ruptures. A fusiform aneurysm is more likely attributed to a dissection aneurysm and carries a higher risk of causing cerebral infarction compared to the classical saccular aneurysm, [25] as was observed also intraoperatively in the first case of our series. Gandhi and colleagues classified aneurysms on the lenticulostriate arteries into two types: type I aneurysms, where the aneurysm dome is not incorporated into the lenticulostriate artery, and type II aneurysms, where the aneurysmal dome is incorporated into the lenticulostriate artery [26].

Notably, the location of the infarctions in our series (basal ganglia, brain stem, cerebellum) and the related terminal artery are similar to the locations of hypertensive intracerebral hemorrhage and the involved vessels (lenticulostriate arteries, thalamoperforators, and paramedian branches of the basilar artery). The fact that the majority of the patients in the present study were of advanced age, and shared the common risk factor of arterial hypertension with those of spontaneous intracerebral hemorrhage, suggests a common pathomechanism likely related to small artery disease [27, 28].

**Fig. 5:**
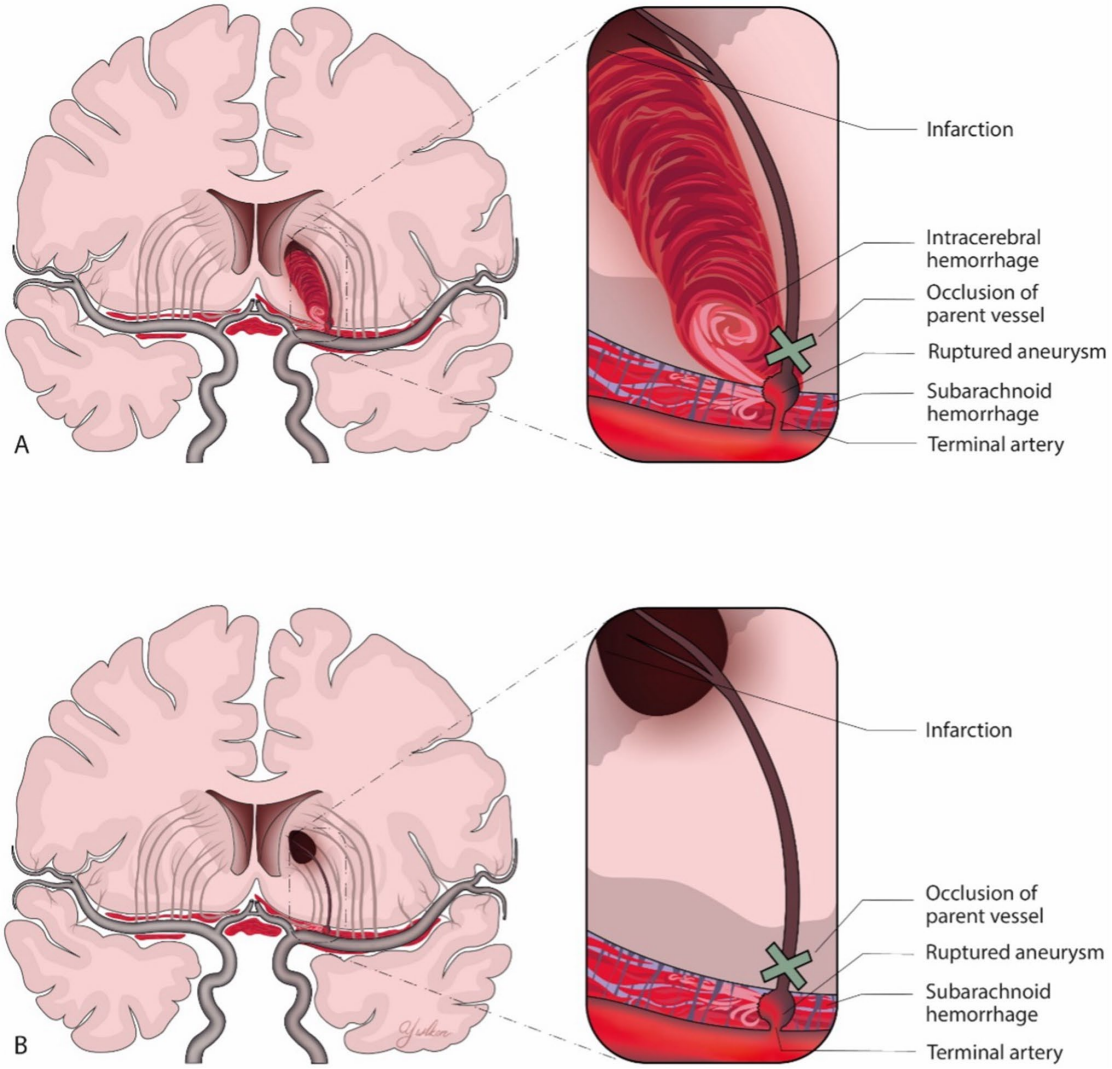
Schematic depiction of the rupture of a lenticulostriate artery aneurysm resulting in concurrent hemorrhage and infarction. (**A**) The rupture of the aneurysm results in both subarachnoid hemorrhage and intracerebral hemorrhage. (**B**) The rupture of the aneurysm results in subarachnoid hemorrhage only in addition to the infarction

Only the location of the rupture in these small vessels determines the type of bleeding: a rupture within the parenchyma leads to intraparenchymal bleeding, while a rupture of an associated aneurysm in the subarachnoid space before the vessel enters the parenchyma results in subarachnoid hemorrhage (Fig. 5). In some cases, a combination of both bleeding types may occur when the rupture is at the interface of the parenchyma and subarachnoid space, as shown in two cases in the present study and also as reported previously (Fig. 5) [24, 29].

Roccatagliata et al. documented angiographic evidence of lenticulostriate artery aneurysms associated with intracerebral hemorrhage to the basal ganglia [30]. Another manifestation of small artery disease is the occlusion of the small terminal artery without rupture, leading to a lacunar infarction without hemorrhage [31].

We also suggest an analogy between Charcot–Bouchard aneurysms in patients with a history of arterial hypertension and the aforementioned aneurysms of a terminal artery. Charcot–Bouchard aneurysms, also known as miliary aneurysms or microaneurysms, are small aneurysms that arise from arterioles usually less than 300 µm in diameter [32]. They were first described by Charcot and Bouchard in 1868 as a cause of hypertensive hemorrhage when they rupture [32].

The higher incidence of intracerebral hemorrhage compared to SAH as a manifestation of small artery disease might be due to structural differences in the intraparenchymal artery segments. These segments are finer, thinner, and have more bifurcations, [22] making them more prone to rupture than those located in the subarachnoid space. Additionally, the subarachnoid segments of these arteries are shorter than the intraparenchymal segments.

Consistent with the results of our study, non-perimesencephalic SAH presents a more aggressive clinical course, with a higher rate of complications and a longer hospital stay [5–8]. As expected, patients diagnosed with non-perimesencephalic SAH showed higher Fisher grades, [33] which is consistent with the observation that patients with a more severe clinical presentation and a larger volume of blood in the subarachnoid space generally have a poorer prognosis [34].

Lenticulostriate arteries aneurysms are rarely documented [14, 23, 24, 35]. They are typically small, with an average maximum diameter of 3.1 mm (range 1–6 mm) [30]. Several conditions have been associated with lenticulostriate artery aneurysms, including Moyamoya disease, and arterial hypertension. Isolated cases have also been reported in association with vasculitis, fibromuscular dysplasia, systemic lupus erythematosus, cocaine use, and brain arteriovenous malformations [30]. The clinical presentation varied depending on the location of the aneurysm within the lenticulostriate artery territory. Distal aneurysms were more frequently associated with focal intracerebral hemorrhage and neurological deficits, while proximal aneurysms were more commonly associated with SAH. There was also a significant overlap in presentation, with distal aneurysms presenting with both intraventricular and intracerebral hemorrhages [29]. In a meta-analysis of 85 lenticulostriate artery aneurysms, 40 (47%) presented with intracerebral hemorrhage, 34 (40%) with intraventricular hemorrhage, and 32 (38%) with SAH, with several patients presenting with more than one type of hemorrhage [29].

Basilar artery perforator aneurysms are characterized by aneurysms where the neck is situated entirely on a perforating artery, without involving the basilar trunk itself [36]. Since Ghogawala et al. first described them in 1996, [37] there has been a rise in reported cases of SAH caused by ruptured of basilar artery perforator aneurysms. Identifying their rupture on digital subtraction angiography is particularly challenging, often requiring repeated angiographies to confirm the diagnosis [38]. Other studies have reported that basilar artery perforating aneurysms can lead to angiogram-negative SAH and may spontaneously disappear [21].

In a review of 29 conservatively treated basilar artery perforating aneurysms, the rebleeding rate was 14.3% [21]. Spontaneous thrombosis of basilar artery perforator aneurysms was also reported [21]. The higher rate of spontaneous disappearance in basilar artery perforating aneurysms, compared to the common saccular aneurysms, may be attributed to the fact that these aneurysms arise from very small perforating arteries of the basilar artery, with a mean diameter of 1.86 mm [21]. As described above, another explanation is that this form of aneurysm is more likely to be a dissecting fusiform aneurysm, which increases the tendency for spontaneous closure of the parent artery. Documented variations in aneurysm size observed in closely repeated angiograms in other studies support the suggestion that these aneurysms are secondary to dissection [21]. Pontine infarction is a documented complication associated with basilar artery perforator aneurysms, occurring in 17.9% (5 out of 28 cases) of the conservatively treated group. Therefore, basilar artery perforating aneurysms as well can cause both hemorrhage and infarction [21].

While a repeat angiogram is likely not necessary in patients with perimesencephalic subarachnoid hemorrhage, as they are usually non-diagnostic and patients tend to have a better prognosis, non-perimesencephalic hemorrhage has been postulated to be more frequently associated with an underlying pathology [3]. Therefore, repeat angiograms are recommended in such cases. Remarkably, however, in our series, the repeat angiogram was negative in all instances, and the question of whether a repeat angiogram is necessary when occlusion of the parent artery due to infarction is detectable remains open. At the time being, we continue to recommend performing a second control angiography in all patients with non-perimesencephalic hemorrhage [39]. Otherwise, all patients with SAH underwent CT angiography during the initial assessment to detect the presence of an aneurysm before being scheduled for digital subtraction angiography. This approach proved useful in detecting ruptured aneurysms in three patients in whom DSA was negative.

Our findings emphasize the importance of considering ruptured aneurysms and occlusion of terminal arteries as potential causes of angiogram-negative, non-perimesencephalic SAH. These patients may require more aggressive monitoring and management than those with perimesencephalic hemorrhage, given the higher likelihood of complications.

This study has several limitations. First, it is retrospective in nature with a relatively small number of patients, which limits the possibility of statistical analysis and generalizability. Second, no patient had pre-hemorrhage MRI, which makes it impossible to fully exclude preexisting infarcts. In addition, not all patients underwent MRI during their clinical course, and small infarcts may therefore have been missed. Third, although the infarcts were localized, singular, and early—likely due to parent artery occlusion after aneurysm rupture—they cannot be distinguished with absolute certainty from vasospasm-related infarcts in all patients. Finally, MRI of the brain and cervical spine was also not obtained routinely in all patients with angio-negative SAH but was performed based on specific neurological signs, or suggestive blood distribution patterns, which may have led to underdetection of small infarcts or spinal sources.

## Data Availability

The data that support the findings of this study are available on request from the corresponding author (S.A.A.)
